# [Corrigendum] New application of an old drug: Antitumor activity and mechanisms of doxycycline in small cell lung cancer

**DOI:** 10.3892/ijo.2025.5754

**Published:** 2025-05-14

**Authors:** Sheng-Qi Wang, Bo-Xin Zhao, Yuan Liu, Ya-Tian Wang, Qian-Ying Liang, Yun Cai, Yun-Qi Zhang, Jiang-Hong Yang, Zhi-Hua Song, Guo-Feng Li

Int J Oncol 48: 1353-1360, 2016; DOI: 10.3892/ijo.2016.3375

Subsequently to the publication of the above paper, an interested reader drew to the authors' attention that, in Fig. 3 on p. 1356 showing the results of cellular apoptosis experiments, the 'Doxy 5 *µ*g/ml' and 'Doxy 10 *µ*g/ml' data panels appeared to contain overlapping data, such that these images were apparently derived from the same original source where the results of differently performed experiments were intended to have been shown. In addition, in Fig. 4A on p. 1357, showing the results of TUNEL assay experiments, the 'Doxy 10 *µ*g/ml' and '5-FU 25 *µ*g/ml' data panels contained overlapping sections, similarly suggesting that these data had been derived from the same original source.

The authors were able to consult their original data, and recognized how these errors occurred. The corrected versions of Figs. 3 and 4, now showing the correct data for the 'Doxy 5 *µ*g/ ml' experiment in Fig. 3 and the '5-FU 25 *µ*g/ml' experiment in Fig. 4A, are shown below and on the next page. The authors regret the errors that were made during the compilation of the original figures, and are grateful to the editor of *International Journal of Oncology* for allowing them the opportunity to publish this Corrigendum. Note that the errors that were made in compiling this pair of figures did not have a significant impact on the conclusions reached in this study. All the authors agree with the publication of this corrigendum; furthermore, they apologize to the readership for any inconvenience caused.

## Figures and Tables

**Figure 3:**
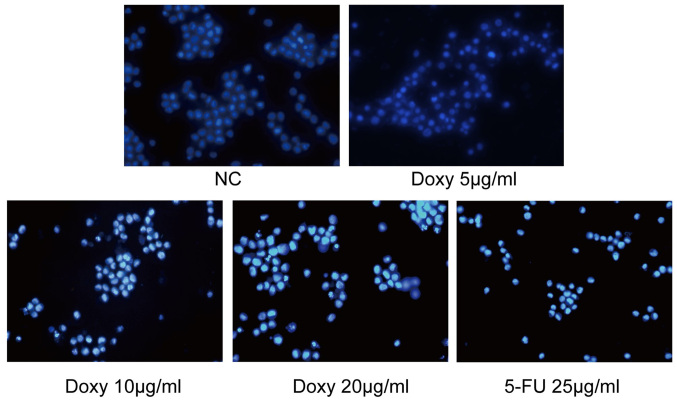
Hoechst 33258 dye staining assay confirmation that doxycycline induces apoptosis of H446 cells.

**Figure 4:**
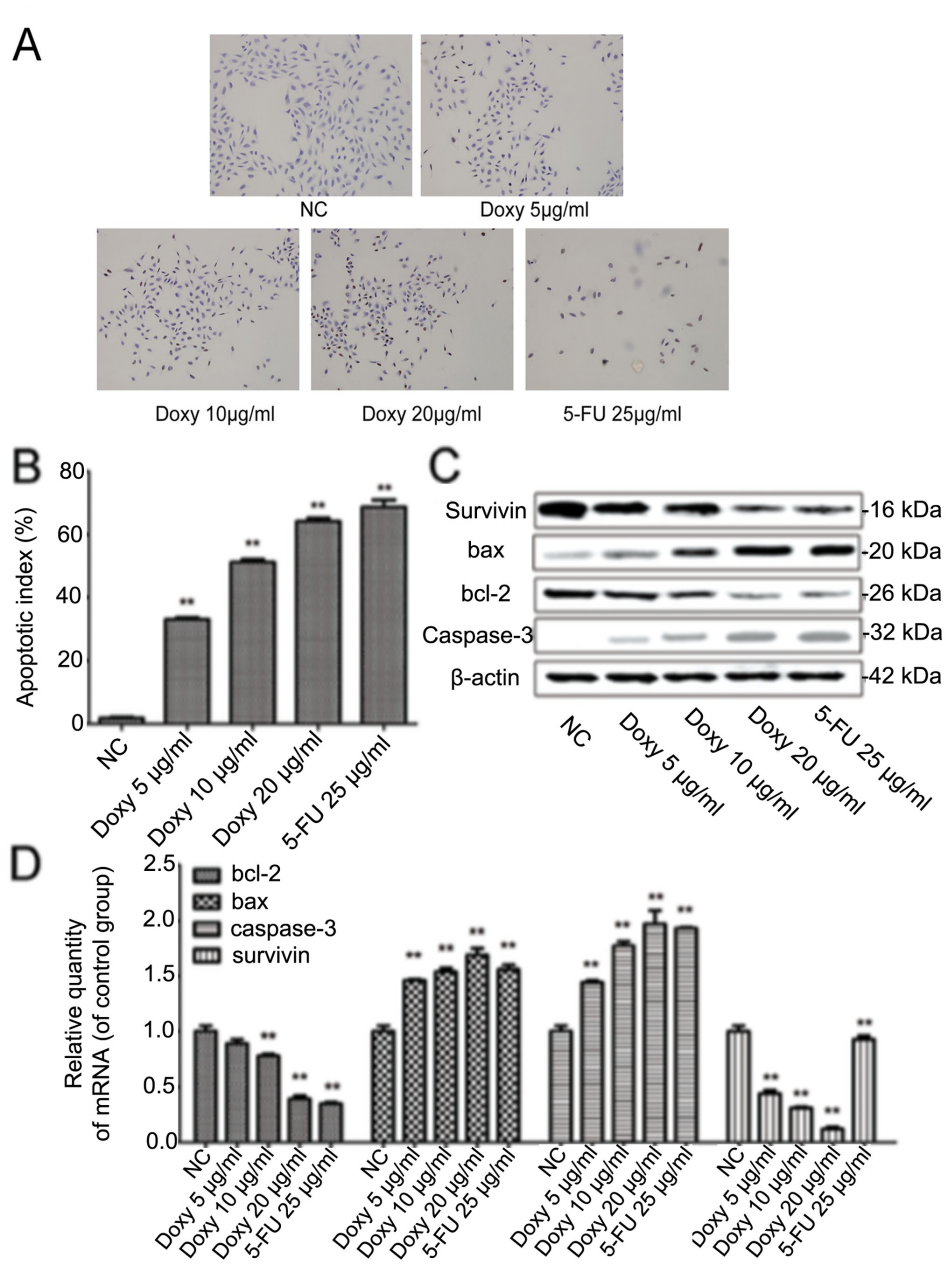
Doxycycline induces apoptosis of H446 cells via regulating the expression of survivin, bax, bcl-2 and caspase-3. (A) Effects of doxycycline on apoptosis of H446 cells were investigated by TUNEL assay; (B) the apoptotic indexes for each group, ^**^p<0.05 vs. control group. (C) Effects of doxycycline on the protein expression of bcl-2, bax, caspase-3 and survivin were investigated by western blot assay; (D) Effects of doxycycline on the mRNA expression of survivin, bax, bcl-2 and caspase-3 were investigated by RT-PCR assay..

